# Does the Biofloc System Affect Water Quality, Reproduction, and Hemato-Immunology of *Penaeus vannamei* During Broodstock Maturation?

**DOI:** 10.3390/ani15233424

**Published:** 2025-11-27

**Authors:** Fernanda Guimarães de Carvalho, Cristhiane Guertler, Felipe Boechàt Vieira, Raphael de Leão Serafini, Haluko Massago, Eduardo da Silva, Jaqueline Inês Alves de Andrade, Edemar Roberto Andreatta

**Affiliations:** 1 Programa de Pós-Graduação em Aquicultura e Recursos Pesqueiros, Laboratório de Camarões Marinhos, Universidade Federal de Santa Catarina (UFSC), Rod. Admar Gonzaga, 1346, Florianópolis 88034-000, SC, Brazil; cristhiane.guertler@ifc.edu.br (C.G.); felipe.vieira@ufsc.br (F.B.V.); edemar.andreatta@ufsc.br (E.R.A.); 2 Laboratório de Aquicultura, Instituto Federal Catarinense Campus Araquari (IFC), BR-280 Km 27, Araquari 89245-000, SC, Brazil; jaqueline.andrade@ifc.edu.br; 3 Departamento de Ensino, Instituto Federal Catarinense Campus Camboriú (IFC), R. Joaquim Garcia, s/n, Centro, Camboriú 88340-055, SC, Brazil; 4 Empresa de Pesquisa Agropecuária e Extensão Rural de Santa Catarina (EPAGRI), Rodovia Antônio Heil, Itaipava, Itajaí 88318-112, SC, Brazil; raphaelserafini@epagri.sc.gov.br (R.d.L.S.); halukomassago@epagri.sc.gov.br (H.M.); 5 Medicina Veterinária, Faculdade Life Unic Education (Life Unic), R. Sen. Felipe Schmidt, 159, Joinville 89201-440, SC, Brazil; eduardo.silva.pr.em@gmail.com

**Keywords:** BFT, marine shrimp, reproductive performance, sustainable aquaculture

## Abstract

Traditional shrimp maturation uses high water exchange, conflicting with sustainable farming and biosecurity, although broodstock production systems have been updated by the adoption of closed-cycle production. So, this study evaluated BFT for *Penaeus vannamei* broodstock maturation, comparing it against a conventional system. The research compared two treatments, BFT and CW, in three replicated tank systems. Each experimental unit was a circular fiberglass tank with 5.63 m^3^ working volume. BFT tanks involved a 3.12 m^2^ mobile triangular cage for viewing and capturing mated females. Parameters monitored included water quality, reproductive performance and hemato-immunological responses. The BFT system resulted in greater water quality stability, significantly lower water consumption, and lower Total Ammonia Nitrogen (TAN) concentrations, indicating efficient ammonia recycling. Also, reproductive performance and most hemato-immunological parameters did not differ significantly between treatments. Although CW showed slightly higher broodstock survival and sperm viability, BFT males exhibited a significantly higher Total Hemocyte Count (THC). Overall, BFT is suitable for sustainable *P. vannamei* broodstock maturation, as it promotes stable environmental conditions without compromising reproductive or immunological performance. Further research is recommended, particularly regarding effects of zero-water-exchange on male shrimp and improvements on capture structures.

## Simple Summary

This study evaluated the effects of biofloc technology (BFT) on water quality, reproduction, and hemato-immunological parameters of *Penaeus vannamei* during broodstock maturation. Comparing clear water (CW) and BFT systems, BFT showed greater water quality stability and lower water consumption, while reproductive and immunological performance did not differ significantly between treatments. The results indicate that BFT allows broodstock maturation without compromising performance, providing more stable and sustainable environmental conditions.

## 1. Introduction

Shrimp are among the most profitable species in global aquaculture and the most widely traded seafood worldwide due to characteristics such as a fast and large-scale production cycle, high reproductive performance, rapid growth, and good adaptability to nursery cultivation [1]. In marine shrimp production, among the different stages of the production cycle is captive maturation for larval production, which traditionally is characterized by low stocking density (<10 shrimp·m^2^), high water-exchange rates (>100%), and a high supply of fresh feed [2], which is inconsistent with the concepts of sustainable farming and biosecurity. In recent years, however, broodstock systems have been updated to enhance biosecurity by adopting closed-cycle production, thereby reducing water use and, consequently, the spread and dissemination of diseases [1]. After all, viral pathogens such as White Spot Syndrome Disease, Infectious Hypodermal and Hematopoietic Necrosis Virus, Infectious Myonecrosis Virus, Yellow Head Virus Genotype 1, Taura Syndrome Virus, and White Tail Disease have caused substantial economic losses to the industry [3].

An essential component of a biosecurity strategy is the implementation of emergency plans for quarantine and pathogen eradication, combined with routine monitoring (surveillance) of shrimp stocks in biosecure facilities [1]. Maintaining broodstock and reproductive processes in closed systems helps stabilize the physicochemical parameters of the culture environment, ensuring greater animal comfort and improved growth performance [4]. Within this context, BFT stands out as a promising alternative for closed aquaculture systems [5,6,7]. BFT involves the formation of microbial aggregates induced by the addition of external carbon sources and continuous and vigorous water aeration [8,9,10]. These aggregates, through a heterotrophic process, control the environmental nitrogenous compounds by immobilizing them during the metabolism of carbohydrates used for the formation of cellular proteins for growth. Thus, the combination of low water-exchange rates with gradual recycling of nitrogen compounds can provide the stability and biosecurity required to enhance reproductive performance, including spawning frequency and the number of eggs per female [11].

Moreover, the microbial aggregates present in BFT not only assimilate ammonia derived from feed and fecal waste but also act as probiotics and immunostimulants, thereby strengthening the immunity of cultured organisms [4]. Despite this potential, the effects of BFT on shrimp broodstock during reproduction remain poorly documented. Therefore, this study aimed to evaluate the influence of biofloc technology on water quality, reproduction, and hemato-immunological parameters of the marine shrimp *Penaeus vannamei* under captive maturation conditions.

## 2. Materials and Methods

This study was conducted at the Marine Shrimp Laboratory (LCM/UFSC), 503 Coroas Street, Zip Code 88061-600, Florianópolis, Santa Catarina, Brazil.

### 2.1. Biological Material

A total of 600 broodstock (300 males and 300 females) of *Penaeus vannamei* were obtained from the LCM/UFSC facilities [12]. Selection criteria included an average body weight of 40 g for both sexes, intact appendages, absence of necrotic areas on the exoskeleton, intermolt stage, and intact spermatophores in males.

After screening, shrimp were transferred with dip nets and buckets to reproduction tanks at a density of 8 shrimp.m^2^ and acclimated to environmental and dietary conditions for 12 days. Unilateral eyestalk ablation was then performed on females. Broodstock were monitored daily for maturation and copulation. At the end of 44 days, survival was determined by comparing initial and final counts in each tank.

### 2.2. Experimental Design and Environment

Two treatments were tested, each with three replicates: Biofloc (BFT) and clear water (CW). Each experimental unit was a circular fiberglass tank (4 m diameter; 5.65 m^3^ working volume) stocked with 50 males and 50 females.

Tanks were filled with seawater (salinity 33–34 g·L^−1^) and maintained under a controlled photoperiod (12.5 h light: 11.5 h dark) using white fluorescent and yellow incandescent lamps programmed by an analog timer [13]. Water temperature was held in each tank at 28.5 ± 0.5 °C with 1800 W titanium heaters (EASYPLUG™, Pentair Aquatic Eco-Systems, Inc., Apopka, FL, USA) and controlled by a Full Gauge thermostat (MT512 RI, Full Gauge Controls, Canoas, Rio Grande do Sul, Brazil).

### 2.3. Feeding Management

During acclimation, broodstock received a standard diet at 3% of biomass per day [14], consisting of: squid (*Loligo* sp., Pioneira da Costa Inc., Porto Belo, Santa Catarina, Brazil), 42% of dry matter; mussel (*Perna perna*, Marine Mollusk Laboratory/UFSC, Florianópolis, Santa Catarina, Brazil), 28%; and commercial feed (Inve Aquaculture—BREED-S FRESH, Fortaleza, Ceará, Brazil—40% crude protein, 9.1% lipid), at a daily rate of 30% of dry matter.

The items were divided into seven feedings at alternating times throughout the day. The amounts were monitored and adjusted weekly for all replicates according to mean body weight, the survival rate and the amount of remaining feed by direct observation of the CW tanks or in two feeding trays placed in the BFT tanks, where the alternate feed portions were equally offered [15].

### 2.4. Biofloc Inoculation

After acclimation, CW tanks received a daily 200% water exchange following LCM/UFSC protocol [16]. On the other hand, BFT tanks were inoculated with 3 m^3^ of biofloc from the laboratory’s nurseries. The inoculum showed the following proximal composition: PO_4_–P 128 mg·L^−1^, total ammonia nitrogen (TAN) 0.2 mg·L^−1^, N–NO_2_ 0.1 mg·L^−1^, and total suspended solids 302 mg·L^−1^. After inoculation, the microbial aggregates were maintained through the animals’ own feeding and the addition of sugar cane molasses, in order to establish a total carbon-nitrogen (C:N) ratio of 20:1 for removal of nitrogen in form of ammonia from the medium [7]. This proportion was measured and maintained taking into account the bromatological compositions of the molasses itself [17] and each item of the adopted diet [18,19].

The suspended material was monitored with the aid of a 40 L settling tank [20], maintained with minimal water exchange and salinity was corrected by pumping fresh water. Aeration was continuous in both treatments: two airlifts arranged in a circular pattern in CW tanks and six airlifts plus a central aeration ring (AeroTubes™, Brasil Piscis, Bofete, São Paulo, Brazil) in BFT tanks.

### 2.5. Broodstock Capture and Handling

In the CW treatment tanks, mature females were identified and recorded by direct observation and captured with dip nets when copulation was detected. In the BFT treatment tanks, however, water clarity was reduced by the presence of microbial flocs. To overcome this limitation, a movable structure was constructed to concentrate and elevate the broodstock, allowing them to be raised to a level that enabled accurate visual inspection.

This device consisted of a triangular cage whose vertices extended from the center of the tank bottom and followed the circumference of the tank. The cage was built entirely of PVC piping. The side pipes had an external diameter of 32 mm, while the main vertex pipe measured 70 mm in diameter so that it could fit over the central drain, thereby preventing broodstock escape. The cage edges were 1.8 m in length, and the cage height was 1 m. One of these edges was detachable at the central vertex, making it mobile and allowing it to be drawn inward to corral the broodstock for capture. This movable edge measured 1.8 m in length and 1.0 m in height. Both the cage and the movable section were covered with hexagonal plastic mesh with a 2 cm aperture.

**Figure 1:**
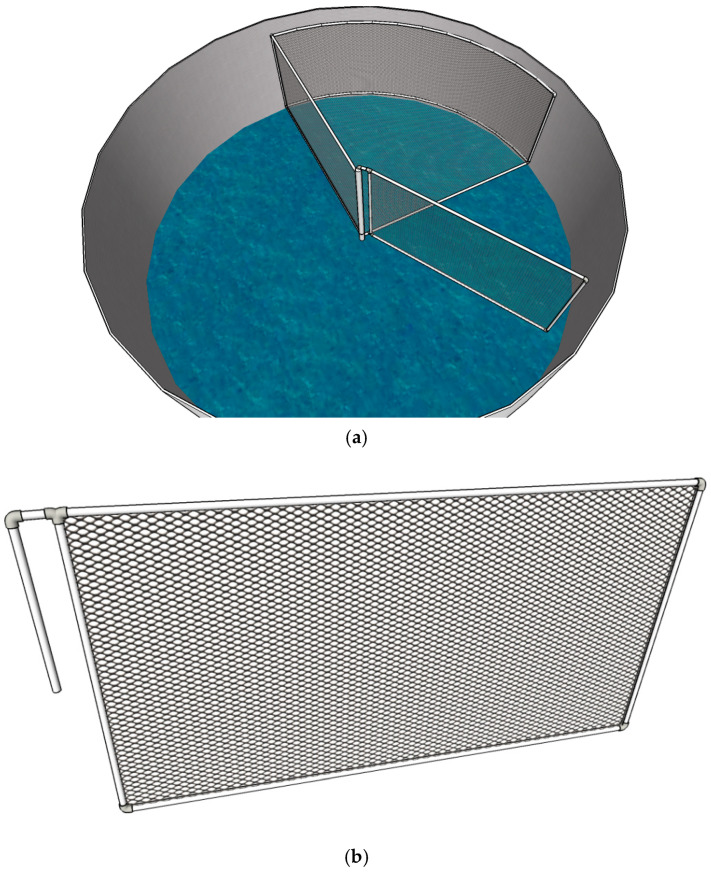
Schematic of the movable cage: complete cage in the tank (**a**); movable gate (**b**) used in *Penaeus vannamei* maturation tanks to capture broodstock for the BFT treatment.

Ropes anchored to the ceiling supported a pulley system used to lift the broodstock for observation. The complete structure in each tank occupied an area of 3.12 m^2^, corresponding to approximately 25% of the tank bottom (Figure 1).

### 2.6. Reproductive Performance

#### 2.6.1. Egg and Nauplius Production

Daily, mature females were recorded and, when mated, individually transferred to 200 L tanks with seawater (29.5 ± 0.5 °C; salinity 30 mg·L^−1^) for spawning. After 4 h, females were returned to their original tanks. Eggs were siphoned, homogenized, and sampled (three 10 mL samples) for counting and extrapolation to total volume. The next morning, nauplii were quantified by the same method and examined microscopically.

#### 2.6.2. Sperm Viability

After 44 days, nine males without spermatophore melanization per tank were divided into three pools. Spermatophores were extracted by pressure on the terminal ampoule [21] and homogenized in 1 mL sterile marine solution (35 mg·L^−1^) to obtain a fresh sperm suspension [22]. Sperm integrity was assessed by light microscopy (400×) (Digilab DI-521B LED, Piracicaba, São Paulo, Brazil) based on the presence of a spine, counting a minimum of 100 cells per sample using a Neubauer chamber, and expressed as the percentage of sperm cells with a spine relative to the total number of cells observed (spined, spineless, and everted) [21,23].

### 2.7. Hemato-Immunological Parameters

Thirty-six individuals (18 males and 18 females) from each treatment were sampled and organized into three pools of three shrimp per sex. Hemolymph was collected from the ventral sinus with a 1 mL syringe and 13 mm × 0.4 mm needle. For this collection, three pools of three males and three pools of three females per treatment were used.

For total hemocyte count (THC), hemolymph was collected in a fixative solution at a known dilution (4% formaldehyde in modified Alsever’s solution consisting of 336 mM NaCl, 115 mM glucose, 27 mM sodium citrate, 9 mM EDTA, pH 7.2), and THC was estimated using a Neubauer chamber (Kasvi, Pinhais, Paraná, Brazil) [24].

For quantification of intracellular superoxide anion production (ROIs), hemolymph was collected in an anticoagulant solution (400 mM NaCl, 100 mM glucose, 30 mM sodium citrate, 10 mM EDTA, 26 mM citric acid, pH 5.5), and NBT (nitro-blue tetrazolium) reduction was performed, adapted from the method of Guertler et al. [25]. Analyses were conducted in quintuplicate.

For hemolymph collection and serum preparation, three pools of three shrimp per sex per treatment were used. After collection, the hemolymph was left at room temperature for 2 h to allow coagulation. The clot was repeatedly macerated and centrifuged at 6000× *g* for 10 min. The serum containing plasma and cellular factors was collected and stored at −20 °C for subsequent analysis.

Agglutinin/lectin titer analysis were performed in duplicate. 50 µL of serum were serially diluted in TBS (50 mM Tris, 5 mM MgCl_2_, 10 mM CaCl_2_, 150 mM NaCl, pH 7.4) in 96-well U-bottom plates and incubated for 2 h at room temperature with an equal volume of 2% canine erythrocyte suspension. The titer was expressed as the reciprocal of the highest dilution showing visible agglutination.

Serum protein concentration was determined by the Bradford method [26] with bovine serum albumin (BSA) as standard, in triplicate. For Phenoloxidase activity, 50 µL of serum (triplicate) were diluted 1:15 in TBS and pre-incubated for 5 min with an equal volume of trypsin (1 mg·mL^−1^, Sigma-Aldrich^®^, São Paulo, Merck KGaA, São Paulo, Brazil). Then, 50 µL of L-3,4-dihydroxyphenylalanine (L-DOPA, 3 mg·mL^−1^) were added. Dopachrome formation was measured by absorbance at 490 nm at 5, 10, 15, and 20 min. One unit of activity was defined as a change of 0.001 Abs min^−1^ mg^−1^ protein at 20 °C [27].

### 2.8. Statistical Analysis

Agglutinin/lectin titer data were log_2_(x + 1)-transformed, and percentages were arcsine-transformed prior to analysis [28]. Data on reproductive performance, water quality, feed intake, and hemato-immunological parameters were subjected to *t*-tests, considering *p* < 0.05 significant. Hemato-immunological parameters by sex and treatment were analyzed by ANOVA followed by *t*-tests (*p* < 0.05) for mean comparisons [29]. *t*-tests and ANOVA were performed using Statistica SystemGVP 2.0.0.

## 3. Results and Discussion

### 3.1. Physicochemical Parameters of Water Quality

**Figure 2:**
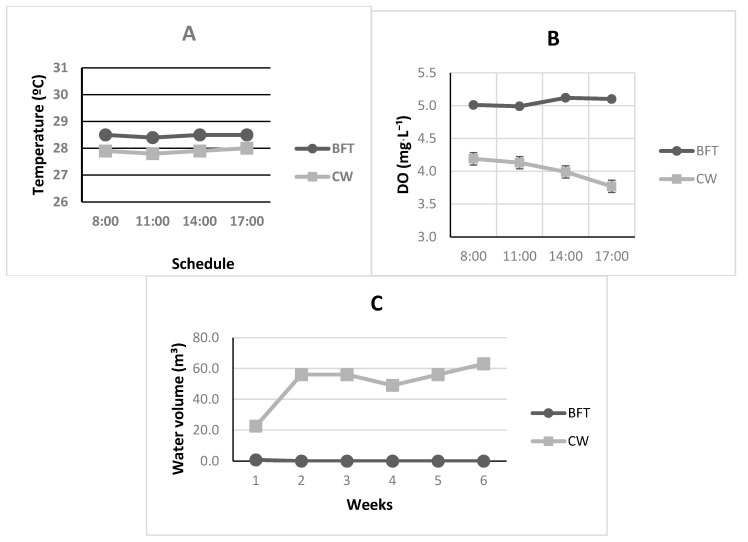
Effects of daily physicochemical water parameters, including Temperature (**A**) and Dissolved Oxygen (**B**), as well as Weekly Water Consumption (**C**), on *Penaeus vannamei* maturation tanks subjected to experimental treatments: Biofloc (BFT) and Clear Water (CW).

Significant differences were observed in almost all physiochemical parameters of water quality (Table 1; *p* < 0.05). As shown in Figure 2, the BFT treatment not only exhibited a significantly lower weekly water consumption (Figure 2C—*p* < 0.0001) but also demonstrated greater stability across all evaluated physical–chemical variables, which supports reproductive events in captivity [30] and makes these organisms less susceptible to stress.

**Figure 3:**
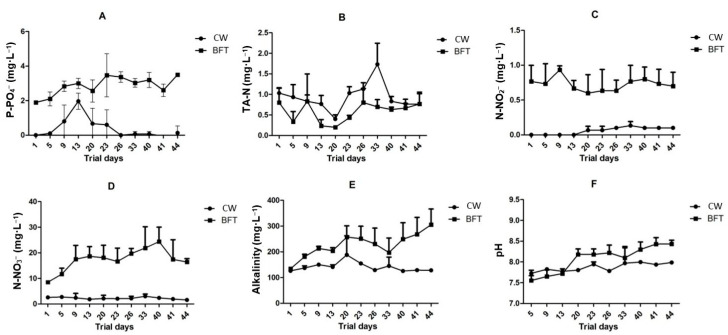
Effects of weekly physicochemical water parameters, including Phosphate (**A**), TAN (**B**), Nitrite (**C**), Nitrate (**D**), Alkalinity (**E**) and pH (**F**), on *Penaeus vannamei* maturation tanks subjected to experimental treatments: Biofloc (BFT) and Clear Water (CW).

Significant differences were observed in alkalinity results (Table 1 and Figure 3E—*p* = 0.0012). Probably due to denitrification process, associated with the characteristic intense heterotrophic microbial activity related to BFT, alkalinity levels in BFT treatment were higher and more variable than those observed in CW [7,9,10]. Despite significant differences found between treatments, all results remained within acceptable levels for the species [31,32,33,34,35].

To ensure maximum profitability in general shrimp production systems, it is essential to achieve the highest efficiency in input utilization [31,32]. Probably due to the daily 200% water exchange, following standard protocol in CW tanks, in this study the BFT treatment showed a significantly lower weekly water consumption (Table 1—*p* < 0.0001). Moreover, no water renewals were necessary in the BFT culture tanks, with water usage solely due to occasional replacement from evaporation during the experimental period. Such significantly lower water volumes not only reduce costs associated with pumping and conditioning water for cultivation but may also enhance system biosafety and sustainability. Reduced water use helps prevent potential pathogen introduction into culture environments and lowers the organic matter discharged into the natural environment.

Significant differences were also observed in temperature results (Table 1 and Figure 2A—*p* < 0.0001). For ectothermic organisms such as marine shrimp, the rate of metabolic and reproductive events is directly proportional to the surrounding temperature [35]. Similar to findings reported by Mugwanya et al. [36], Otoshi et al. [34], and González-González et al. [37] in their recirculation studies, water temperature in the BFT systems remained more stable throughout the day when compared with the CW treatment during the experimental period (Figure 2A). This outcome is likely also related to the standard renewal rates used in CW treatment [30]. Nevertheless, both treatments reached average values suitable for captive maturation of the species [31].

Surprisingly, a similar pattern was observed for dissolved oxygen (DO) data, where the BFT treatment showed a significantly better and more stable levels than in CW tanks (Table 1 and Figure 2B—*p* < 0.0001). These results, while indicating the effectiveness of the system applied in BFT treatment, also suggest a possible need for improvements in aeration system of CW tanks of this study. Madenjian et al. [38] note that DO is one of the most critical parameters for cultured species, and levels below 2 mg·L^−^^1^ can cause severe stress or even mass mortality in confined organisms. For captive reproduction, Barbieri Jr. and Ostrenski Neto [2] add that DO should be maintained above 5.0 mg·L^−^^1^, comparable to natural marine conditions. Therefore, as shown in Table 1, the BFT results fall within the recommended levels for the species, whereas the CW values were below the advised range. Gandhi et al. [33] reported mean concentrations of 5.76 ± 0.51 mg·L^−^^1^ for this parameter in their recirculating system for *Farfantepenaeus aztecus* reproduction, while Otoshi et al. [34] found concentrations of 6.5 and 6.4 mg·L^−^^1^ in captive breeding experiments with *P. vannamei* in recirculating systems.

TSS, transparency, phosphate, nitrite, and nitrate levels in BFT replicates were significantly higher than those observed in CW (Table 1—*p* < 0.0001). Krummenauer et al. [39], Menasveta et al. [40], and Colt [41] explain that this discrepancy is expected and results from the low or absent water exchange in closed systems, which allows nitrogen compounds and phosphate to accumulate in the culture environment. Arana [42] notes that nitrite, an intermediate in the nitrification process, has a characteristically high affinity for copper present in the hemolymph. Its presence therefore leads to the formation of met-hemocyanin, which is unable to transport oxygen and can cause death by asphyxiation in confined organisms.

However, Lin and Chen [43] determined that a safe nitrite level for *P. vannamei* juveniles is 25.7 mg·L^−1^, since higher salinity reduces the compound’s toxicity [38]. Conversely, Van Rijn et al. [44] reported that nitrate has low toxicity for aquatic organisms, while Wickins [45] observed that a phosphate concentration of 94 mg·L^−1^ caused no mortality in *Macrobrachium rosenbergii*. Thus, although statistically different, both treatments remain within acceptable ranges for the species [43,44,45].

Unexpectedly, mean TAN concentrations were lower in the BFT treatment than in CW (Table 1—*p* = 0.012). Likewise, as shown in Figure 3B, this trend persisted throughout the experimental period, with the CW treatment reaching peak TAN concentrations on day 33. In addition, TAN levels were less variable in the BFT treatment over time.

High concentrations of this compound can exceed the organisms’ excretory capacity, leading to its accumulation in the hemolymph. Such accumulation, in turn, causes gill lesions, reduces oxygen-carrying capacity, lowers circulating pH, induces histological damage to blood cells, interferes with respiratory processes, and increases susceptibility to disease [39,40,42,43]. For captive maturation systems of penaeid shrimp, levels between 2.6 and 4.2 mg·L^−1^ are recommended. Both treatments remained within the maximum acceptable range for the species [40,42,43]; however, these results indicate the high efficiency of the microbial community established in BFT treatment in ammonia recycling, as well as a universe of possibilities for biosecure and sustainable reproductive environments in captivity.

### 3.2. Reproductive Performance

Aside from sperm viability (*p* = 0.0047) and broodstock survival (*p* = 0.0001), no significant differences were observed between treatments for percentage of mature females and percentage of mature and copulated females; number of eggs (×10^3^) and nauplii (×10^3^) and percentage of hatching rate, except for a slight superiority in the CW treatment (Table 2). Despite CW showing somewhat higher results than BFT, both treatments exhibited satisfactory performance regarding sperm viability [46,47,48]. In their evaluation of age and weight effects in male *P. vannamei*, Ceballos-Vazquez et al. [49] reported results ranging from 12.8% to 68.2% for males aged 6–12 months, whereas Perez-Velazquez et al. [50] obtained values between 0% and 63.3% when assessing the effect of different temperatures on *P. vannamei* sperm quality. These findings are therefore lower than those obtained in the present study.

A significant difference was detected between treatments with respect to survival, with broodstock in the CW treatment achieving better results than those in the BFT treatment. However, it should be noted that the survival rates can be considered low for this species in both treatments [51]. These outcomes are likely associated with intrinsic aspects of the environment and the management of captive maturation [52], as well as with physiological events involved in gonadal maturation [32].

González-González et al. [37] explain that the reproductive performance of captive penaeid shrimp can be influenced by environmental factors such as temperature, dissolved oxygen, and nitrogen compounds, as well as by internal factors including origin, nutritional status, and/or genetics. Because the broodstock used in both treatments of this study share the same origin and showed no significant differences in most of the zootechnical indices evaluated, the low reproductive performance likely stems from internal factors associated with these broodstock [31].

Specifically, regarding the percentage of mated mature females compared with sperm viability performance, Alfaro-Montoya et al. [48] explain that captive maturation often enhances reproductive performance; however, it is possible that males are particularly sensitive to zero-exchange systems. This supports the theory that ambient temperatures above 27 °C negatively affect the reproductive performance of captive male penaeid shrimp [48,50], suggesting a potential need for separating males and females and adopting dedicated mating tanks to meet these gender-specific requirements.

### 3.3. Hematological–Immunological Parameters

#### 3.3.1. Total Hemocyte Count (THC)

Significant differences were detected between sexes and between treatments for this parameter (Table 3). Males subjected to the BFT treatment showed higher values than all other groups. Ekasari et al. [53] and Xu et al. [54] observed a clear effect of the presence of bioflocs on the immune system of penaeid shrimp. They theorized that the broad variety of microorganisms characteristic of bioflocs environments could stimulate hemocyte proliferation in the hemolymph of penaeid shrimp. That evidence, associated with a presumable male greater sensibility to zero-exchange systems, may explain these results.

THC values in females of both treatments were close to the penaeid standard described in the literature. Perazzolo et al. [55] reported values ranging from 17.9 to 22.8 × 10^6^ cells·mL^−1^ for *F. paulensis* subjected to eyestalk ablation or spermatophore removal, while Sainz-Hernández et al. [56] obtained values between 15 and 26 × 10^6^ cells·mL^−1^ for *P. vannamei* subjected to unilateral or bilateral ablation. Le Moullac and Haffner [57] demonstrated a positive correlation between ambient temperature and THC, and the same pattern was observed in males of *L. setiferus* [58]. Thus, the slight superiority observed in the BFT treatment can be attributed to the higher temperatures maintained in the culture environment during the present study.

#### 3.3.2. Quantification of Intracellular Superoxide Anion Production (ROIs)

No significant differences were found between treatments. CW males showed the lowest values, whereas CW females showed the highest (Table 3). Despite the absence of significant differences, high baseline levels were observed in both treatments and sexes. The presence of elevated ROIs across all treatments suggests stress conditions that may be linked to physiological events related to reproduction [59], particularly in captivity, rather than to the applied treatments.

Furthermore, the nutritional profile of the broodstock diet likely did not offset this chronic stress [59]. Finally, the large variability in reference values, the range of factors that can interfere with hemato-immunological profiles, and the lack of standardization in the technical evaluation of these indices [60,61,62,63] limit the precision of possible inferences from these analyses.

#### 3.3.3. Agglutinin/Lectin Titer

No significant difference was found for this parameter. Additionally, the high values recorded (Table 3) were close to the averages reported for the reproductive phase of penaeids [60,61,62]. Although these results indicate a stress condition associated with physiological events related to captive breeding, rather than the treatments applied, they also suggest an adequate immune response in both treatments [62,64,65].

#### 3.3.4. Serum Protein Concentration (PC)

Again, no significant difference was noted between treatments (Table 3). Perazzolo et al. [55] observed protein concentration reductions in adult *F. paulensis* exposed to salinity changes, ablation, and spermatophore removal, reporting values between 60 and 70 mg·mL^−1^, while Racotta and Palacios [66] reported decreases in hemolymph protein levels in *P. vannamei* under stress.

However, the values obtained in this study can be considered high compared with previously reported standards for penaeid shrimp. Palacios et al. [67] observed a slight positive correlation between the number of spawnings and hemolymph protein concentration in both wild and captive *P. vannamei* females, but their values remained below 150 mg·mL^−1^, whereas Maggioni et al. [28] reported 260–308 mg·mL^−1^ when testing ascorbic acid overdosing in broodstock diets, similar to the present findings. Finally, Sánchez et al. [59] suggested that hemolymph protein concentration may be linked both to hemocyanin content—about 90% of total hemolymph proteins—and to the organism’s reserve proteins, reflecting the crude protein level in the diet of the analyzed shrimp.

#### 3.3.5. Phenoloxidase Activity (PO)

No significant difference was detected between treatments (Table 3); however, for both genders, the values observed in CW treatment were higher than those in BFT. As noted above, reference values for hemato-immunological parameters are highly variable within the same population, sex, or developmental stage. The literature reports several external factors that may alter PO levels in captive maturation environments, including fluctuations in nitrogen compound concentrations in the culture medium [47,57], hypoxia [57,68], unilateral ablation [51,56], and handling stress [62,66]. This can be explained by the physiological changes and energy expenditure inherent to reproduction, which generate considerable stress in cultured organisms regardless of the specific captive-maturation system adopted.

The high values observed in this study (Table 3) indicate that broodstock may have been affected by some stressors. Schleder et al. [69] reported increased PO activity in sexually mature *Nodipecten nodosus*, while Sánchez et al. [59] observed the same in reproductive males of *L. setiferus* subjected to acclimation handling between 27 °C and 31 °C. These authors also note that one possible effect of stress is a reduction in the organism’s immune potential through decreased circulating hemocytes and altered regulatory mechanisms of PO, which in turn elevates PO activity. For both sexes, THC levels were higher in BFT, likely due to the effect of biofloc presence on the immune system of these organisms. Therefore, this may have been reflected in the observed PO activity results.

## 4. Conclusions

Neither the BFT nor the CW treatment produced significant effects on the reproductive performance or the immunological profile of the broodstock evaluated. Additionally, BFT treatment showed better performance than CW treatment related to most water quality physicochemical parameters analyzed in the present study. Future studies are needed to clarify the effects of zero-water-exchange systems on male shrimp and to develop improved capture structures. Nevertheless, the results presented here suggest that biofloc technology can be applied to captive maturation without compromising the reproductive or immunological performance of marine shrimp *Penaeus vannamei*.

## Figures and Tables

**Table 1:** Results of water physicochemical parameters (mean ± s.e.) in *Penaeus vannamei* maturation tanks subjected to experimental treatments: Biofloc (BFT) and Clear Water (CW).

Parameter	Treatment	
	CW	BFT
Temperature (°C) ^†^	27.87 ± 0.04	28.46 ± 0.01 ^†^
Dissolved Oxygen (mg·L^−1^) ^†^	4.00 ± 0.03	5.10 ± 0.03 ^†^
Phosphate (mg·L^−1^) ^†^	0.39 ± 0.18	2.87 ± 0.16 ^†^
Nitrite (mg·L^−1^) ^†^	0.06 ± 0.01	0.72 ± 0.03 ^†^
Nitrate (mg·L^−1^) ^†^	2.27 ± 0.12	17.35 ± 1.31 ^†^
Ammonia (mg·L^−1^) ^†^	0.93 ± 0.09	0.58 ± 0.07 ^†^
Alkalinity (mg·L^−1^) ^†^	141.00 ± 5.59	226.50 ± 14.20 ^†^
pH	7.87 ± 0.03	8.07 ± 0.10
Weekly water consumption (m^3^) ^†^	50.00 ± 5.90	0.13 ± 0.10 ^†^
TSS (mg·L^−1^) ^†^	0.00 ± 0.0	390.60 ± 18.44 ^†^
Transparency (cm) ^†^	45.00 ± 0.0	20.10 ± 0.20 ^†^

^†^: significant difference (*p* < 0.05).

**Table 2:** Reproductive performance (mean ± s.e.) of marine shrimp *Penaeus vannamei* broodstock submitted to experimental treatments: Bioflocs (BFT) and Clear Water (CW).

Parameter	Treatment	
	CW	BFT
Survival (%) ^†^	67.00 ± 1.20 ^†^	42.00 ± 1.20
% mature females	5.59 ± 0.74	4.36 ± 0.72
% mature and copulated females	18.00 ± 3.80	9.90 ± 3.50
Eggs (×10^3^)	48.00 ± 7.80	34.00 ± 11.00
Nauplii (×10^3^)	21.00 ± 6.30	16.00 ± 5.60
Hatching rate (%)	34.00 ± 4.70	43.00 ± 5.20
Sperm viability (%) ^†^	95.00 ± 0.67 ^†^	91.00 ± 0.78

^†^: significant difference (*p* < 0.05).

**Table 3:** Total hemocyte count (THC), superoxide anion quantification (ROIs), agglutinating activity, serum protein concentration (PC) and phenoloxidase activity (PO) (mean ± s.e.) of marine shrimp *Penaeus vannamei* broodstock submitted to experimental treatments: Biofloc (BFT) and Clear Water (CW).

Parameter	Males		Females	
	CW	BFT	CW	BFT
THC (10^6^ cels·mL^−1^) ^†^	30.2 ± 1.61	41.11 ± 0.19 ^†^	25.08 ± 1.63	29.81 ± 3.39
ROIs (OD_630_)	0.67 ± 0.02	0.89 ± 0.01	1.14 ± 0.006	1.08 ± 0.02
Agglutinin/lectin titer (log_2_)	15.25 ± 0.58	14.58 ± 0.00	15.25 ± 0.58	15.58 ± 0.00
PC (mg·mL^−1^)	289.24 ± 0.47	289.37 ± 0.83	289.53 ± 0.50	291.29 ± 2.65
PO (U.min^−1^·mg^−1^) ^†^	45.50 ± 1.80 ^†^	35.12 ± 2.47	40.57 ± 5.14	38.29 ± 1.74

^†^: significant difference (*p* < 0.05).

## Data Availability

The data presented in this study are available on request from the corresponding author.

## Abbreviations

The following abbreviations are used in this manuscript:

BFT	Biofloc Technology
LCM	Laboratório de Camarão Marinho—Marine Shrimp Laboratory
UFCS	Universidade Federal de Santa Catarina
CW	Clear Water
THC	Total Hemocyte Count
ROI	Superoxide Anion Production
BSA	Bovine Serum Albumin
DO	Dissolved Oxygen
TSS	Total Suspended Solids
TAN	Total Ammonia Nitrogen
PC	Protein Concentration
PO	Phenoloxidase Activity
